# External Oblique Intercostal Block for Living Kidney Donor Open Nephrectomy: A Case Series

**DOI:** 10.7759/cureus.39139

**Published:** 2023-05-17

**Authors:** Catarina Petiz, Rita Barbosa, Teresa Ribeiro Boneco, Jânia Pacheco, Alexandra Resende

**Affiliations:** 1 Anesthesiology, Centro Hospitalar Universitário Lisboa Norte, Lisboa, PRT

**Keywords:** loco-regional anaesthesia, pain, external oblique intercostal block, open nephrectomy, case report

## Abstract

The external oblique intercostal (EOI) block is a novel regional technique that provides analgesia for upper abdominal incisions. We performed single-injection and continuous EOI blocks in living kidney donors who underwent open nephrectomy. In this case series, we report our experience with pain management using this technique in five patients at our centre. EOI block resulted in good pain relief in our patients. The median (IQR) numerical rating scale score was 3 (1-6) at rest immediately after the end of the surgery, predominantly visceral. We want to highlight the benefits regarding pain management of the association of EOI block with conventional therapy.

## Introduction

Donor nephrectomy is a unique surgery where an otherwise healthy individual is subjected to the hazards of a major procedure entirely for altruistic purposes. Live kidney donation is an important alternative for patients with end-stage renal disease. Conventional open living-donor nephrectomy is performed under routine general anaesthesia and is associated with prolonged postoperative pain. Multimodal, rather than opioid-only, analgesia is generally preferable. The epidural catheter is an effective method of analgesia and is described in this type of surgery. However, unfractionated heparin may be required just prior to the renal artery occlusion [1]. In the event of vessel puncture with a bloody tap during the neuraxial technique, full intra-operative heparinization should be avoided for six to 12 hours [2]. Therefore, neuraxial anaesthesia should be rethought. An alternative to this technique is the external oblique intercostal (EOI) plane block. It was described and explained by Elsharkawy et al. in 2021 and provides analgesia to the anterior and lateral abdominal wall in dermatomes T7-T11 [3]. It is a simple and effective block and provides analgesia of the upper abdominal wall with minimal side effects. A catheter can be inserted in the EOI plane, which allows pain control in the postoperative period [4]. Other advantages include easy sonoanatomy (even in obese patients), being performed in the supine position, and no anticoagulation concern [3]. In this case series, we report our experience with pain management using the EOI block in five kidney donors at our centre.

## Case presentation

**Figure 1:**
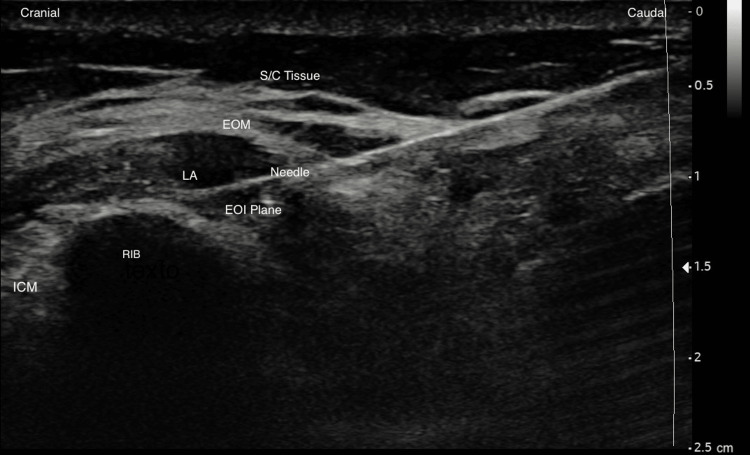
Ultrasound image of the external oblique intercostal plane block ICM: intercostal muscle; EOI: external oblique intercostal; EOM: external oblique muscle; S/C: subcutaneous; LA: local anaesthetic.

Written informed consent was obtained from all the patients in this report. We describe the use of EOI in our anaesthetic approach of five living kidney donors who underwent open nephrectomies. The five patients, whose ages ranged from 30 to 50 years, were classified as American Society of Anesthesiologists (ASA) grade I-II (Table 1). General anaesthesia induction was performed with fentanyl, propofol, and rocuronium and maintained with sevoflurane. The block was performed at the end of the surgery, with the patient still under anaesthesia. All blocks and catheter placement were performed by a specialized anaesthetist using the technique described by Elsharkawy et al. [3]. With the patient positioned in the supine position with their ipsilateral arm abducted, an ultrasound scan using a high-frequency linear transducer (13-6 MHz, Sonosite SII Stand, FUJIFILM Sonosite, Inc., Bothell, WA) was performed. The skin was sterilized, and the probe was placed over the sixth rib medial to the anterior axillary line in a parasagittal orientation. A SonoLong Echo (Pajunk, Geisingen, Germany) - echogenic catheter - through the needle was used. The needle was inserted in a cephalocaudal direction, and the external oblique intercostal plane was hydrodissected with saline. After hydrodissection, the needle was advanced 1 to 2 cm, and the catheter was placed another 4 cm beyond the tip of the needle under ultrasound guidance. The catheter was then secured, and 20 ml of 0.2% ropivacaine was administered. Correct placement of the catheter was confirmed by the ultrasound visualization of the local anaesthetic dispersion in the desired location (Figure 1). Further analgesia was supplied with intravenous paracetamol 1 g and metamizole 2 g. Each patient was extubated at the end of the anaesthesia after neuromuscular block reversion and then transferred from the operating room (OR) to the post-anaesthetic care unit (PACU). Before discharge from the PACU, an anaesthesia provider assessed and examined each patient to ensure patient safety before transfer to the ward. The post-operative analgesia included patient-controlled regional analgesia (PCRA) with 20 ml ropivacaine 0.2% every four hours, supplemented by 10 ml bolus in SOS (with one-hour lock-out), following institutional protocol. Also, paracetamol 1 g 8/8h, metamizole 1 g 8/8h, and tramadol 100 mg in SOS were prescribed. During their recovery time in the hospital, our team provided multiple visits to register patients' pain evaluations. Pain assessment was achieved using the numerical rating scale (NRS). NRS scores were registered at rest and movement immediately after surgery and at two, six, 12, 24, 36, 48, 60, and 72 hours after surgery. The need for analgesic rescue was also registered. If still in place, catheters were removed by the acute pain team 72 hours after the technique was performed.

**Table 1:** Patients' characteristics ASA: American Society of Anesthesiologists.

Patients' characteristics					
Patient	ASA	Age	BMI (kg/m^2^)	Habits	Comorbidities
						1	I	50s	27.8	Former smoker	0
2	II	30s	26	Smoker	Dyslipidemia
3	II	30s	23	Smoker	0
4	II	50s	25	Smoker	0
5	II	30s	29.4	Smoker; sporadic cocaine consumption	Anxiety

Case 1

The patient was in their 50s with a BMI of 27.8 kg/m^2^ with a history of smoking (10 pack-years). The patient had no known diseases and was not currently taking any ambulatory medication. Pre-operative exams were conducted, and no significant alterations were found. The individual was donating to their daughter. EOI block and catheter were positioned following protocol, without any complications. No pain in the immediate postoperative period was reported. The patient complained of pain two hours after admission to the PACU (NRS = 3, predominantly visceral), which responded to 100 mg of intravenous tramadol with pain relief (NRS = 1). Satisfactory pain relief while in the ward was reported, and the patient described dermatomal sensory coverage of T7-T11. On the second and third days, the patient described pain at movement (NRS = 5). However, the patient was not resourcing to PCRA manual bolus of ropivacaine.

Case 2

The patient was in their 30s with a BMI of 26 Kg/m^2^ with a history of dyslipidemia and smoking (five pack-years). The patient was not currently taking any ambulatory medication and pre-operative exams did not show any significant alterations. The individual was donating to their daughter. EOI block and catheter were positioned without complications following protocol. Pain in the immediate postoperative period was reported (NRS at rest = 6, parietal and visceral characteristics), with relief after administration of 100 mg of IV tramadol and ropivacaine bolus through the catheter (NRS at rest = 3). The patient had satisfactory pain relief in the ward, with no need for SOS therapy. The patient reported sensory coverage in the dermatomes of T7-T11. Fifty hours after surgery, the catheter was accidentally exteriorized, with a new onset of parietal pain with movement (NRS = 5 with movement).

Case 3

The patient was in their 30s with a BMI of 23 kg/m^2^ with a history of smoking (two pack-years). Pre-operative exams were conducted and no significant alterations were found. The individual was donating to their son. EOI block and catheter were positioned without complications following protocol. There was a need for rescue analgesia after PACU admission with 100 mg of IV tramadol due to visceral and parietal pain (NRS = 3), having achieved pain relief (NRS = 1 at rest). The patient reported good pain relief with resort to PCRA during the ward stay. The patient reported dermatomal sensory coverage of T7-T11.

Case 4

The patient was in their 50s with a BMI of 25 Kg/m^2^ with a history of smoking (15 pack-years). The patient had no other relevant medical history and was not currently taking any ambulatory medication. The individual was donating to their son. EOI block and catheter were positioned without complications following protocol. Two hours after being admitted to the PACU, the patient complained of visceral pain (NRS = 4 at rest), which responded to 100 mg of IV tramadol (NRS = 1). During the stay in the ward, the patient reported satisfactory pain control, with no need for rescue analgesia apart from ropivacaine bolus through the catheter. The patient reported dermatomal sensory coverage of T7-T11.

Case 5

The patient was in their 30s with a BMI of 29.4 kg/m^2^ with a history of smoking (10 pack-year), sporadic cocaine consumption, and anxiety disorder medicated with diazepam. Pre-operative exams were within normal limits. The individual was donating to their cousin. EOI block and catheter were positioned without complications following protocol. Visceral and parietal pain in the immediate postoperative period (NRS = 3 at rest) motivated the IV administration of 100 mg of tramadol and 40 mg of parecoxib as rescue therapy. Six hours post-surgery, the patient reported good pain relief with no need for rescue analgesia. The patient reported dermatomal sensory coverage of T7-T11. The patient reported moderate pain at movement 48 hours after surgery (NRS = 5). NRS is a subjective evaluation and is dependent on individual factors. Apart from that, the patient was avoiding rescue bolus, which might have limited the control of pain.

Overall, all patients reported good pain relief six hours after surgery at rest and movement (Table 2), and deambulation on the second postoperative day was achieved. Following surgery, a single dose of 100 mg tramadol (equivalent to 10 morphine milligram equivalents (MMEs)) was administered to the patients, with no requirement for additional opioid therapy during their ward stay. The pain was subjectively graded on a numeric rating scale of 0 to 10, where 0 represented no pain and 10 represented the worst pain imaginable. The median (IQR) NRS was 3 (1-6) at rest immediately after the end of the surgery, predominantly visceral. The median (IQR) NRS scores after six, 12, 24, and 48 hours were 2 (1-3), 2 (1-1), 0 (0-1), and 0 (0-0), respectively.

**Table 2:** Observed outcomes NRS: numerical rating scale; R: rest; M: movement.

Observed outcomes																	
Patient		NRS0		NRS2		NRS6		NRS12		NRS24		NRS48		NRS60		NRS72	
		R	M	R	M	R	M	R	M	R	M	R	M	R	M	R	M
1		1	2	1	2	1	2	2	4	2	5	3	5	0	1	0	1
2		6	8	3	6	3	3	2	3	1	2	0	1	1	5		
3		7	7	3	3	2	2	1	3	0	2	0	1	0	1	0	1
4		0	0	4	5	3	2	2	1	0	4	0	1				
5		3	3	3	3	0	0	0	1	0	1	0	1	0	1	0	1
Median (IQR)		3 (1-6)	3 (2-7)	3 (3-3)	3 (3-5)	2 (1-3)	2 (2-2)	2 (1-2)	3 (1-3)	0 (0-1)	2 (2-4)	0 (0-0)	1 (1-1)	0 (0-0)	1 (1-2)	0 (0-0)	1 (1-1)

## Discussion

Upper abdominal incisions such as a subcostal oblique laparotomy used for nephrectomies are a cause of intense pain. Epidural analgesia remains the gold standard for major abdominal surgery. However, epidural analgesia is associated with potential side effects, such as hypotension, urinary retention, and extended time to first ambulation post-surgery [5]. Moreover, it is dependent on coagulation status. EOI block is simple to use, is dependable, has a reduced side-effect profile, and is suitable for upper abdominal wall analgesia. It can safely be performed after general anaesthesia induction and is easily done in the supine position. Several fascial blocks have been employed for abdominal surgeries, but they exhibit certain drawbacks. While the transversus abdominis plane block offers analgesia for T10 to L1, it fails to cover the incision site [3,6]. Anterior quadratus lumborum block provides effective analgesia for T7 to L1, but it is technically more challenging and deep. Paravertebral and erector spinae blocks require the patient to be in a non-supine position [7].

Leaving a catheter and PCRA may have several advantages for pain management, such as continuous pain relief and flexibility of pain management, allowing the patient to self-administer small doses of pain medication as needed, giving them more control over their pain management and improving patient satisfaction [8]. PCRA was programmed with 20 ml ropivacaine 0.2% every four hours, supplemented by 10 ml bolus in SOS (with one-hour lock-out). Moving forward, we will continue to monitor the literature for updates on optimal volumes and timing of administration for this technique.

Although the EOI block with a continuous catheter placement produced very positive results, we acknowledge the presence of different outcomes and may try to justify them based on the subjectivity of pain that is dependent on individual differences in physiological, emotional, and cognitive states and unpredictable efficacy of fascial plane blocks. Potential strategies to enhance the effectiveness of fascial plane blocks include precise deposition close to the targeted area, injection of adequate volumes to facilitate physical spread by bulk flow, and manipulation of concentration to encourage diffusion [9].

A disadvantage of this blockade comes from the fact that it does not ensure visceral analgesia, which represented a handicap in our patients, and rescue IV analgesia with tramadol was necessary in the PACU. However, opioids were not needed to control pain in the ward. The administration of a limited amount of opioids (10 MMEs) during the postoperative period suggests that the EOI block was effective in providing analgesia and acted as an opioid-sparing technique.

## Conclusions

This case series provides compelling evidence that continuous EOI block is a suitable method of providing pain relief during the postoperative period of open nephrectomies. EOI block can serve as a substitute in situations where epidural use is contraindicated or technically challenging. However, future studies are needed to further establish the generalizability and robustness of our findings and to compare EOI block to other pain management techniques for open nephrectomy techniques.
